# Splicing stimulates siRNA formation at *Drosophila* DNA double-strand breaks

**DOI:** 10.1371/journal.pgen.1006861

**Published:** 2017-06-19

**Authors:** Karin Merk, Marco Breinig, Romy Böttcher, Stefan Krebs, Helmut Blum, Michael Boutros, Klaus Förstemann

**Affiliations:** 1 Gene Center and Dept. of Biochemistry, Ludwig-Maximilians-Universität München, München, Germany; 2 Division Signaling and Functional Genomics, German Cancer Research Center (DKFZ) and Heidelberg University, Im Neuenheimer Feld 580, Heidelberg, Germany; 3 Laboratory for Functional Genome Analysis (LAFUGA), Gene Center, Ludwig-Maximilians-Universität München, München, Germany; Institute of Molecular Biology, GERMANY

## Abstract

DNA double-strand breaks trigger the production of locus-derived siRNAs in fruit flies, human cells and plants. At least in flies, their biogenesis depends on active transcription running towards the break. Since siRNAs derive from a double-stranded RNA precursor, a major question is how broken DNA ends can generate matching sense and antisense transcripts. We performed a genome-wide RNAi-screen in cultured *Drosophila* cells, which revealed that in addition to DNA repair factors, many spliceosome components are required for efficient siRNA generation. We validated this observation through site-specific DNA cleavage with CRISPR-*cas9* followed by deep sequencing of small RNAs. DNA breaks in intron-less genes or upstream of a gene’s first intron did not efficiently trigger siRNA production. When DNA double-strand breaks were induced downstream of an intron, however, this led to robust siRNA generation. Furthermore, a downstream break slowed down splicing of the upstream intron and a detailed analysis of siRNA coverage at the targeted locus revealed that unspliced pre-mRNA contributes the sense strand to the siRNA precursor. Since splicing factors are stimulating the response but unspliced transcripts are entering the siRNA biogenesis, the spliceosome is apparently stalled in a pre-catalytic state and serves as a signaling hub. We conclude that convergent transcription at DNA breaks is stimulated by a splicing dependent control process. The resulting double-stranded RNA is converted into siRNAs that instruct the degradation of cognate mRNAs. In addition to a potential role in DNA repair, the break-induced transcription may thus be a means to cull improper RNAs from the transcriptome of *Drosophila melanogaster*. Since the splicing factors identified in our screen also stimulated siRNA production from high copy transgenes, it is possible that this surveillance mechanism serves in genome defense beyond DNA double-strand breaks.

## Author summary

DNA damage ultimately threatens the integrity of our genome; in addition, it is an impediment for transcription and thus affects cells independently of the mutagenic effects. DNA double-strand breaks in transcribed regions can induce the production of corresponding small interfering RNAs, a class of regulators that is well known for controlling gene expression. The reason why they are generated at sites of DNA damage is not understood, putative roles in DNA repair remain controversial. Thus, the function of DNA damage-induced siRNAs is a major open question. In an unbiased approach, we screened *Drosophila* cells genome-wide to detect those factors that are required to produce damage-induced siRNAs. In addition to DNA repair proteins, we found that many splicing factors and spliceosome components were necessary. The need for an intron upstream of the break was confirmed by *cas9*-CRISPR mediated cleavage, followed by small RNA sequencing. A detailed look at the siRNA coverage revealed nonetheless that unspliced transcripts give rise to siRNAs. Hence, the spliceosome must be stalled and signal to the transcription machinery. The new role of damage-induced siRNAs in local RNA surveillance should be considered on par with their putative function during DNA repair.

## Introduction

DNA is constantly challenged by mutagenic processes of extrinsic and intrinsic origin. Of these damages, DNA double-strand breaks are particularly problematic lesions because they disrupt the continuity of genetic information. Their repair can either proceed via end-joining activities or through homology-directed repair [1]. A detailed mechanistic understanding of these repair processes is not only important for the prevention and treatment of diseases, such as cancer, but also to help researchers direct the outcome of genome editing experiments with precision [2, 3]. The information stored in DNA is read-out by the process of transcription into RNA. DNA damage is therefore not only an impediment for replication, but also a hindrance for RNA biosynthesis. If DNA damage has occurred in an actively transcribed region, concomitant action of DNA repair factors and the transcription machinery is not possible; access to the DNA must thus be regulated [4]. Stalling of an RNA polymerase upstream of the damaged site may lead to extensive RNA-DNA hybrids, called R-loops, which can themselves cause genomic instability [5–7]. Consequently, a domain of specific chromatin states is assembled around a DNA double-strand break and transcriptional silencing can occur [8]. On the other hand, RNA polymerases stalled by certain types of base damage serve as sensors and thus promote repair of the lesion during transcription-couple repair [9, 10]. It is also established that some RNA binding proteins are recruited to sites of DNA damage [5] and non-coding transcription may play an important role in DNA repair [11, 12]. Finally, the Prp19 component of the spliceosome can interact with RPA bound to single-stranded DNA and reinforces activation of the protein kinase ATR in a manner that is independent of its function during the splicing reaction[13].

Recently, formation of locus-specific siRNAs has been observed at DNA double-strand breaks [14–17]. They may promote repair via homologous recombination in mammalian cells and plants [15, 18], but the molecular mechanisms through which siRNAs promote homologous recombination are not fully established; in mammalian cells they may involve targeting of Rad51 to the damaged site via protein-protein interactions with Ago2 [18], but it is challenging to exclude indirect effects via perturbed miRNA biogenesis in these experiments. In *Neurospora crassa*, their biogenesis is even dependent on, rather than important for, homologous recombination [19–21]. In *Drosophila* the generation of DNA damage-induced siRNAs depends on transcription and is limited to only one side of the broken DNA, the region between a transcription start site and the DNA end. It was thus proposed that the DNA ends serve as transcription initiation sites to generate corresponding antisense transcripts to generate dsRNA [16]. Consistently, transcription initiation at DNA breaks has now been directly observed in *S*. *pombe* [12]. However, no DNA repair defects could be observed in *dcr2* mutant *Drosophila melanogaster* flies where the siRNA pathway is completely inactivated but the miRNA pathway remains mostly unperturbed [22]. Although the significance of the siRNAs for DNA repair is thus a matter of debate, their presence demonstrates that transcripts running towards a DNA DSB are subject to some sort of surveillance and, as a consequence, at least partially converted into double-stranded RNA (the precursor of siRNAs).

To shed light on the mechanistic details of this process, we conducted a genome-wide RNA interference screen in cultured *Drosophila* cells using siRNAs generated from a linearized plasmid. These siRNAs control expression of a reporter gene. In addition to DNA double-strand break repair proteins, we discovered that many splicing factors, in particular components from the Prp19 and Prp19-related spliceosome sub-complexes are important for siRNA generation. Consistently, the presence of upstream introns greatly stimulated siRNA generation at chromosomal DNA DSBs induced by CRISPR-*cas9*. An intron-less gene, on the other hand, only generated few siRNAs upon cleavage. We propose that the perturbed transcript maturation that ensues when RNA polymerase II encounters a DNA double-strand break is sensed with participation of the spliceosome. As a consequence, double-stranded RNA is generated and processed into siRNAs. Intriguingly, the splicing factors identified in our screen for break-induced siRNA generation were also important for siRNA generation from high-copy transgenes. It is thus conceivable that the transcript maturation surveillance mechanism serves in genome defense beyond DNA double-strand breaks.

## Results

### A genome-wide RNAi screen for triggers of DNA double-strand break derived siRNAs

We had previously described a reporter system that allows for a dual luciferase-based readout of DNA double-strand break derived siRNA activity [16]. In short, a linearized plasmid with either a truncated or an inverted coding sequence of *Renilla* luciferase is co-transfected with a mix of circular expression vectors for *Renilla* and firefly luciferase to control for transfection efficiency. The siRNAs generated from the linearized plasmid can then repress full-length *Renilla* luciferase expression *in trans*. When combined with prior experimental RNAi, this assay system has a high signal-to-noise ratio and can easily be scaled up. Note that the promoters in all reporter constructs contain an intron in the 5’-UTR, which precedes the *Renilla* or firefly luciferase CDS. We thus performed a genome-wide RNAi screen in *Drosophila* S2-cells to identify factors that are required for the generation of DNA double-strand break derived siRNAs (see S1 Fig and S2 Fig for details). Two independent biological replicates of the entire screen were performed and averaged.

After removing likely false positives, such as retracted gene models or genes that are not expressed in S2-cells, we selected a total of 142 positive and 66 negative candidates from the screen for further validation (S1 Fig and S1 Table). We re-screened the original dsRNA trigger of our candidates for a third biological replicate and then generated two independent, non-overlapping dsRNAs for each candidate to identify false positives due to off-target RNAi. Only those candidates that scored positive for at least two out of the three distinct RNAi triggers (= screened dsRNA and two validation constructs) were retained. We also counter-screened the entire set of candidates with a cell line where a GFP reporter is repressed by two perfect matches to miR-277 in its 3’-UTR; expression of this reporter is driven by the same promoter as the *Renilla* luciferase in the screen. Since miR-277 is processed by Dcr-1, then loaded into Ago2 via the Dcr-2/R2D2 complex, we could distinguish between core RNAi pathway components or factors that non-specifically activate transcription of our reporter and those factors that are specifically required for DNA damage-induced siRNA biogenesis. After this stringent validation process (summarized in S1 Table), we retained a set of 89 genes that promote DSB-derived siRNA generation or function and 36 candidates that are potential repressors of DBS-derived siRNA production.

### Processing of the DNA ends stimulates siRNA generation

**Fig 1:**
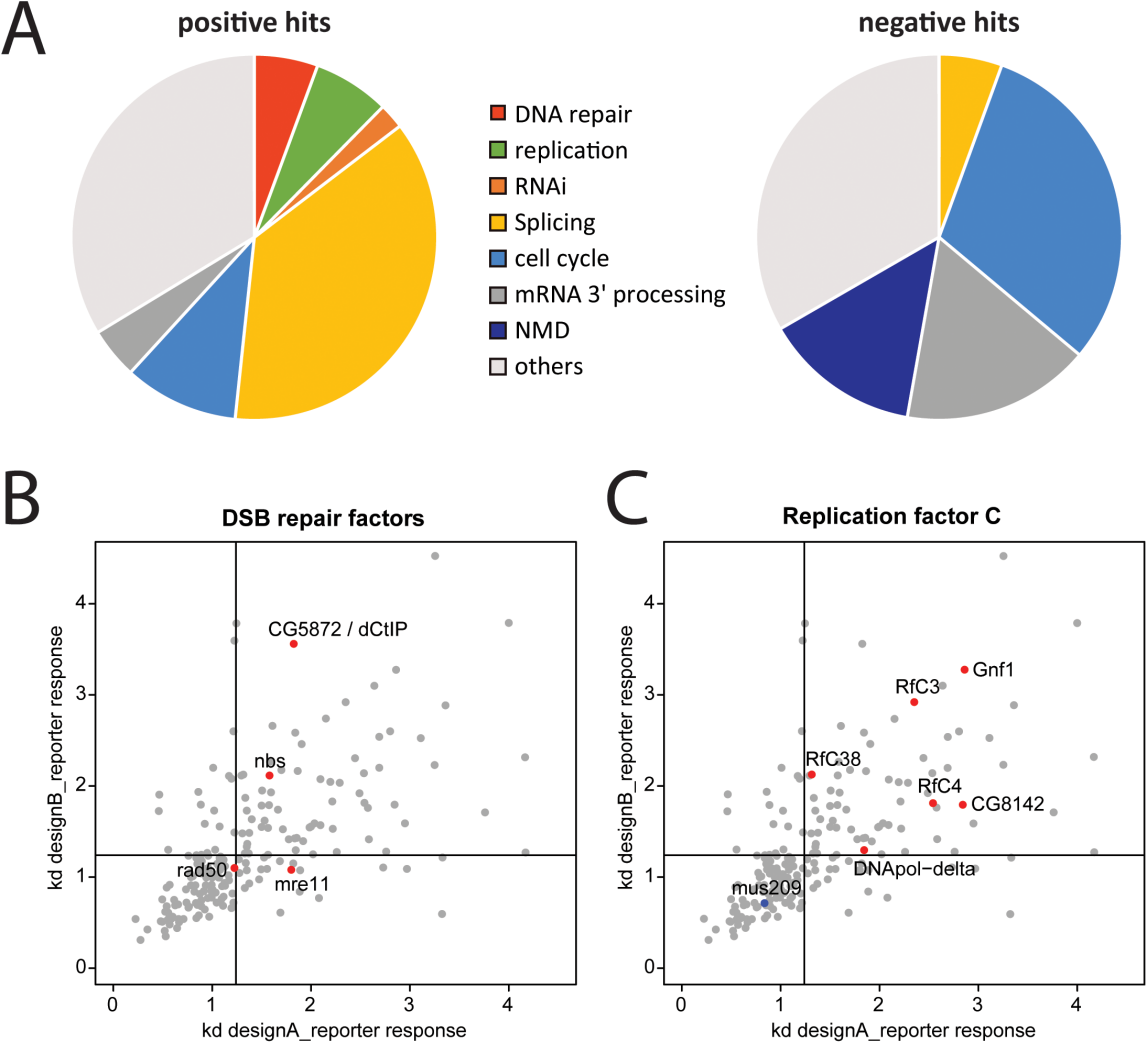
Analysis of the screening data for siRNA induction at a DNA double-strand break. (A) Pie-charts of selected gene ontology terms among the 89 positive and 36 negative candidates obtained after screening and validation; the genes were analyzed with GOrilla [55]. (B) Specific labeling of the MRE11-Rad50-Nbs1 (MRN) complex in the context of the validation dataset. The putative Drosophila CtIP homolog, CG5872, was also recovered and confirmed in our screen. (C) Specific labeling of the replication factor C (RfC) subunits and DNA polymerase δ in the context of the validation dataset. Note that the *Drosophila* PCNA homolog, *mus209*, could not be confirmed as a negative hit in our validation experiments. For (A) and (C), the threshold lines were set at the mean plus 1x standard deviation of our negative controls (knock-down of GFP/DsRed).

To obtain an initial overview of the biological processes involved in siRNA generation at the DNA break we performed a gene ontology analysis of the validated candidates (Fig 1A) and calculated significances using g:profiler [23]. As expected for a screen with linearized DNA, we identified a series of DNA replication/repair factors among the positive candidates (GO-term enrichment of “DNA metabolic process” with a *p*-value of 9.0x10^-5^). DNA double-strand breaks are recognized by the Mre11-Rad50-Nbs1 (MRN) complex; all components of this complex were among the initial candidates and 2 out of 3 passed our validation experiments. In addition to DNA damage signaling, the MRN complex initiates 5’ to 3’resection of the break and thus commits the site for homology-directed repair. The 3’ single-stranded end is subsequently extended further by CtIP/Sae2, promoting homologous recombination [24]. Bioinformatic analysis has suggested that the *Drosophila* CtIP homolog is CG5872 [25] and this gene was also required for correct re-localization of a heterochromatic DSB [26]. We identified CG5872 as a strong candidate in our screen; CG5872 is thus most likely the *Drosophila* homolog of CtIP/Sae2 and we propose that it should be called *dCtIP* (Fig 1B). For further repair, DNA synthesis carried out by the replicative polymerases DNA-polδ and DNA-polε after Rad51-mediated annealing of the exposed 3’ single-stranded regions with a homologous template. We identified DNA-polδ and all subunits of replication factor C (RfC) as stimulators of break-induced siRNA generation. RfC normally loads the processivity clamp proliferating cell nuclear antigen (PCNA, called *mus209* in *Drosophila*) to ensure long-range DNA synthesis. However, the PCNA-homolog *mus209* was initially among the negative candidates but did not pass our validation criteria (Fig 1C). NHEJ factors, such as the Ku70/Ku80 complex, were not identified in the screen. Taken together, our screening efforts demonstrate that recognition and processing of the DNA double-strand break for homology-directed repair promotes and thus precedes siRNA generation.

### Splicing stimulates siRNA generation

**Fig 2:**
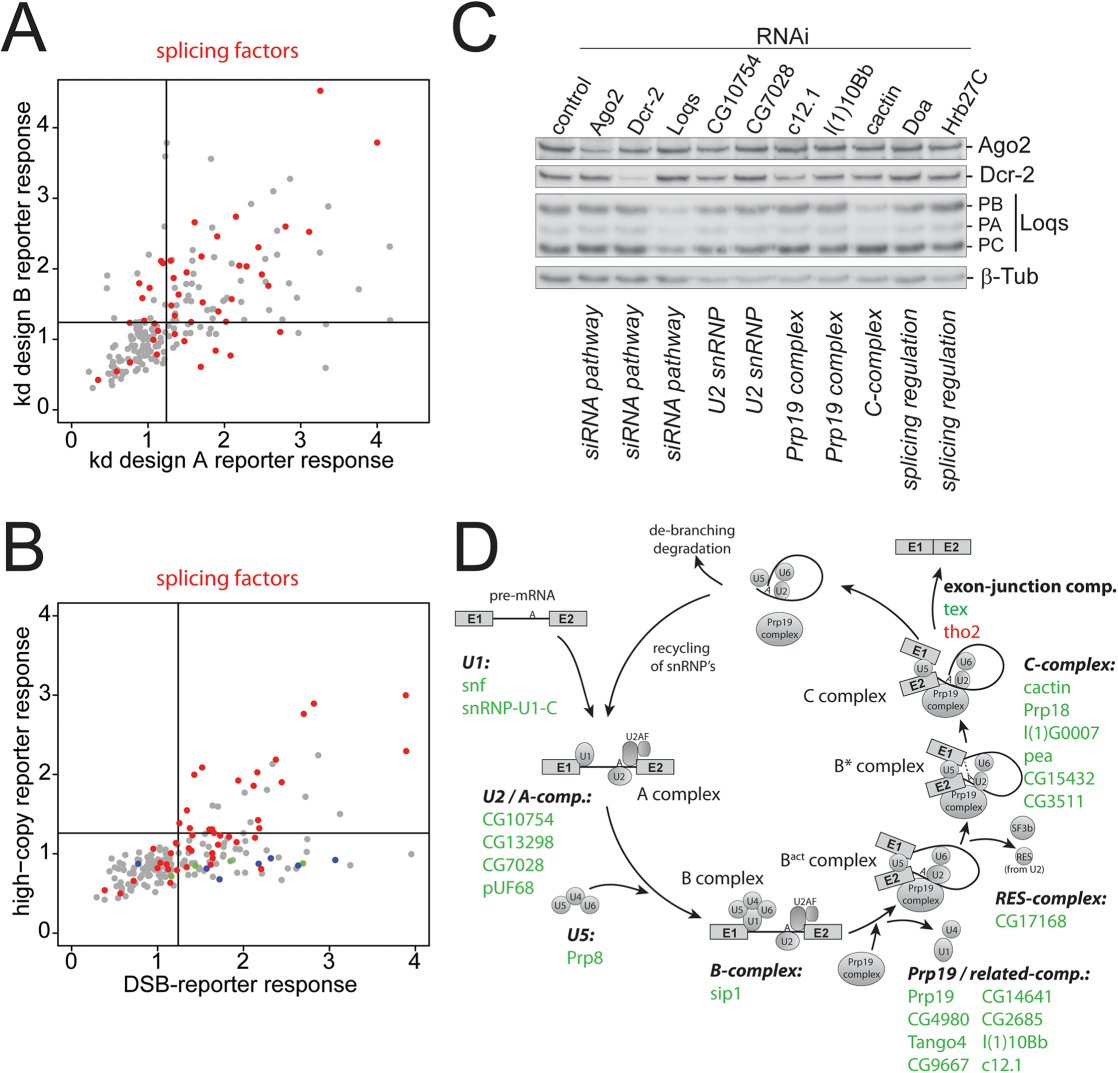
Analysis of splicing factors recovered in the screen. (A) All factors with the GO-term annotation “splicing” were labeled in red in the context of the validation dataset. These factors had a similar distribution with respect to effect strength and validation success as other candidates. (B) Direct comparison of the effect strength obtained with splicing factors on DNA DSB induced siRNAs (abscissa) and high-copy transgene induced siRNAs (ordinate). Splicing factors are marked in red, while DNA repair factors and RfC components (see Fig 1) are marked in green and blue, respectively. The full dataset is contained in S1 Table. (C) Western Blot analysis to determine whether knock-down of splicing factors leads to impaired expression of core RNAi components. The knock-down is indicated above each lane, the monoclonal antibody used for detection is indicated on the right. All RNAi antibodies were kind gifts of Dr. Mikiko Siomi, the β-tubulin loading control was obtained from the developmental studies hybridoma bank. (D) A map of all spliceosome components identified in the screen onto the various complexes that assemble along the course of a splicing reaction. See also S3 Fig for more detail.

Much more striking than the recovery of the DNA repair factors, however, was the enrichment of splicing factors (see Fig 1A, positive candidates). For example, the GO-term “RNA splicing” was found enriched among the candidates with a *p*-value of 2x10^-29^. Among the potential repressors of DSB-derived siRNAs, we found that mRNA 3’-end processing activities were enriched (e.g. GO-term “mRNA cleavage” *p*-value 4.7x10^-14^). Although GO-term analyses must be interpreted with caution, this overview is consistent with the hypothesis that DSB-derived siRNAs are generated with a contribution of the mRNA splicing reaction. On the other hand, canonical transcript termination via mRNA cleavage appears to remove the trigger for siRNA generation. The candidates with the GO-term association “splicing” showed a validation success rate comparable to other candidates (Fig 2A). Since the involvement of splicing is reminiscent of a recently proposed model for transposon recognition in the fungus *Cryptococcus neoformans* [27], we tested our candidates for their requirement to repress a genomically integrated, endo-siRNA generating high-copy transgene analogous to a previously published system [28]. In the current study, we measured a cell line where the high-copy integrated *Renilla* luciferase responds more strongly to an impaired RNAi pathway than the firefly luciferase integrated at low copy-number; since identical plasmid constructs were used this reporter system allows for direct comparison with the screening data (Fig 2B). This comparison defined two groups of candidates: The first is required for the generation of DNA break induced as well as high-copy transgene induced siRNAs and comprises, among others, the splicing factors. The second group is specific for DNA double-strand break induced siRNAs and comprises the homologous recombination factors discussed above (including dCtIP) as well as RfC. Perturbed mRNA splicing may thus be a common trigger for siRNA biogenesis at DNA double-strand breaks as well as high-copy transgenes.

On the other hand, RNAi-mediated depletion of splicing factors could indirectly affect siRNA biogenesis; for example, altered splicing efficiencies could result in lower protein levels of core RNAi factors. Our analysis with the miR-277 perfect match reporter cell line suggested that upon knockdown of the splicing factors, the core RNAi pathway is unaffected and reporter expression is unchanged (S1 Table). In addition, we tested several candidates involved in the splicing reaction for de-regulation of RNAi factors via Western blot and saw no consistent protein level changes for core RNAi components (Fig 2C). We constructed a map of our candidates on the various spliceosome complexes that assemble along the path of a splicing reaction (Fig 2D). Although we identified factors from all complexes, the recovery was particularly prominent among members of the Prp19- and Prp19-related complexes (8 out of 16 vs. 26 out of 138 for all spliceosome components, *p*<0,04 χ^2^-test, see also S3 Fig). This complex has a pivotal role in enabling the transitions from the pre-catalytic spliceosome into the catalytic phases.

**Fig 3:**
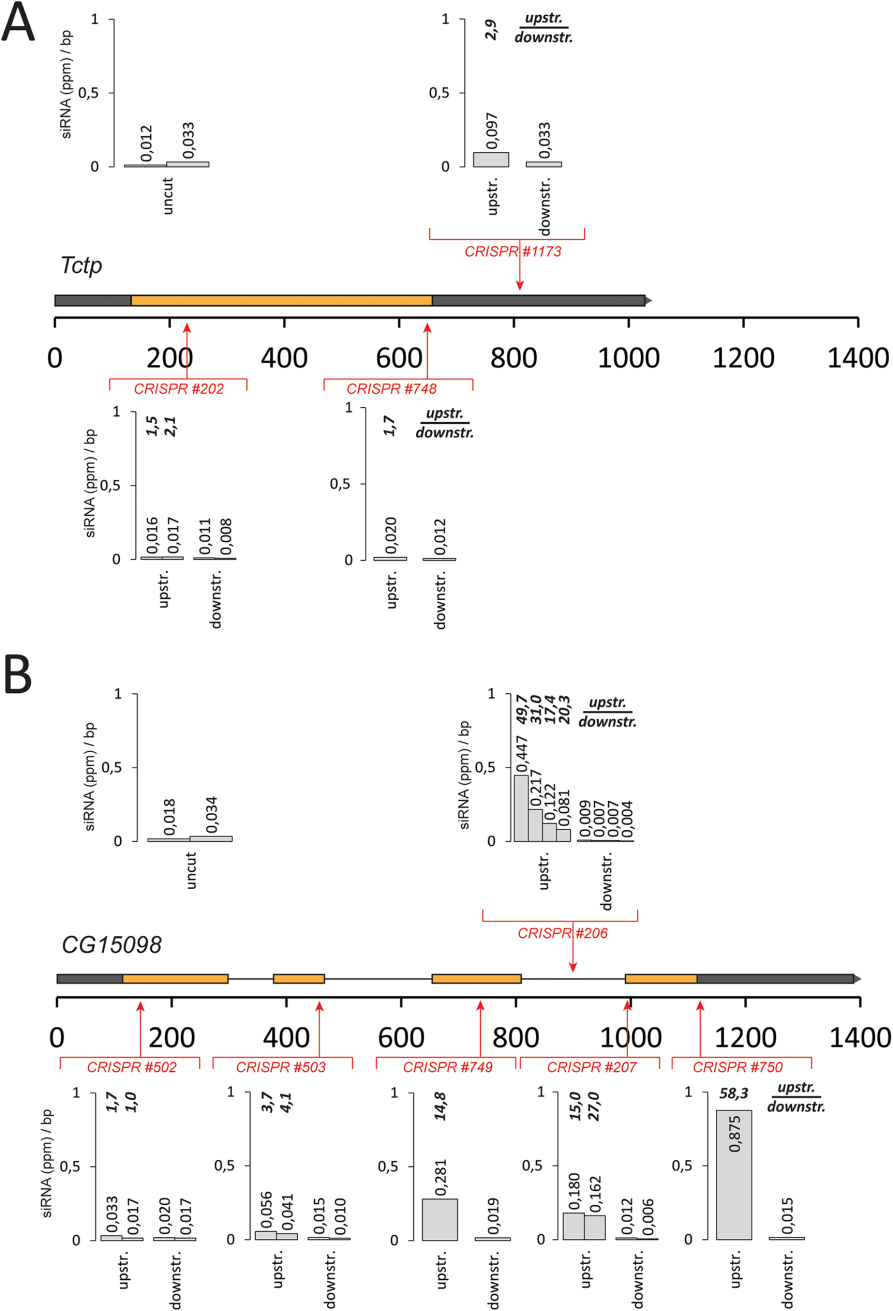
Correlation between gene structure and efficiency of DSB-induced siRNA formation. We calculated the normalized read frequency (reads per million) and determined the per base average for the region between the transcription start site up to the cut (“upstream”, excluding the region where reads derived from the sgRNA itself map) and for the region between the cut until the annotated transcript 3’-end (“downstream”). Detailed traces of siRNAs mapping to the respective loci are provided in S5 Fig (TCTP) and 6 (CG15098). (A) cas9-CRISPR cuts in the intronless *tctp* gene result in barely any production of siRNAs. (B) Cuts in the intron-containing gene *CG15098* can efficiently produce DNA-damage dependent siRNAs, provided that the cut is sufficiently downstream of an intron.

We wanted to confirm the importance of splicing for break-induced siRNA generation by creating DNA breaks at specific positions relative to splice sites. To this end, we employed the *cas9*-CRISPR nuclease technology and programmed the enzyme to cleave chromosomal sites before or after an intron, then deep sequenced the small RNAs and mapped them back to the cleaved locus. To obtain a quantitative measure of siRNA generation at a given site, we calculated the normalized read density per base pair of the affected transcript upstream and downstream of the cleavage site. If break-derived siRNAs are efficiently generated, then the average reads/bp values are higher between the promoter and the DSB site than in the region following the break [16]. We first targeted a strongly expressed, intron-less gene (*tctp*) for cleavage at three different positions. Although cleavage at each site was detectable (as judged by a T7 endonuclease assay, S4 Fig), we observed siRNA generation that was only slightly above background (average ratios of before vs. after the cut of 1.6–2.9, Fig 3A). Analogously, when we targeted the spliced gene CG15098 (with a similar expression level) before the first intron, we observed only low levels of siRNA generation (Fig 3B). A cut close to the first intron-exon junction (82 nt downstream) resulted in rather moderate siRNA induction. Cleavage further downstream, however, led to a strong generation of siRNAs with an increasing ratio of reads upstream vs. downstream of the break (up to 58.3) as the cut was moved downstream along the gene (Fig 3B, see also S5 Fig and S6 Fig for detailed traces). Thus, splicing of the transcript affected by the DNA break greatly stimulates siRNA generation.

We extended our analysis to the CG18273 gene, which is only moderately expressed in S2-cells (S7 Fig). Upon *cas9*-mediated DSB induction, an siRNA response could be induced here as well. Interestingly, this gene showed moderate, cleavage-independent siRNA coverage in its 3’-portion. Prior to this zone, the DNA-break induced coverage was about 10-fold lower than the coverages we observed in the cleaved CG15098 gene. The strength of the DSB-induced siRNA response correlates thus with the host gene expression level, consistent with the notion that the mRNA transcript contributes the sense strand to dsRNA formation. CG18273 has a short (60 nt) first intron followed by a rather long second exon (2375 nt). Cleavage within this second exon resulted in siRNA formation, indicating that a single short intron can suffice to trigger the response. Furthermore, when we induced DNA cleavage close to the end of the CG18273 gene (4686 nt downstream of the transcription start site), we observed siRNA coverage all the way to the start of the transcriptional unit. The DSB-induced small RNA response can thus cover a window of several kbp even in moderately expressed regions.

**Fig 4:**
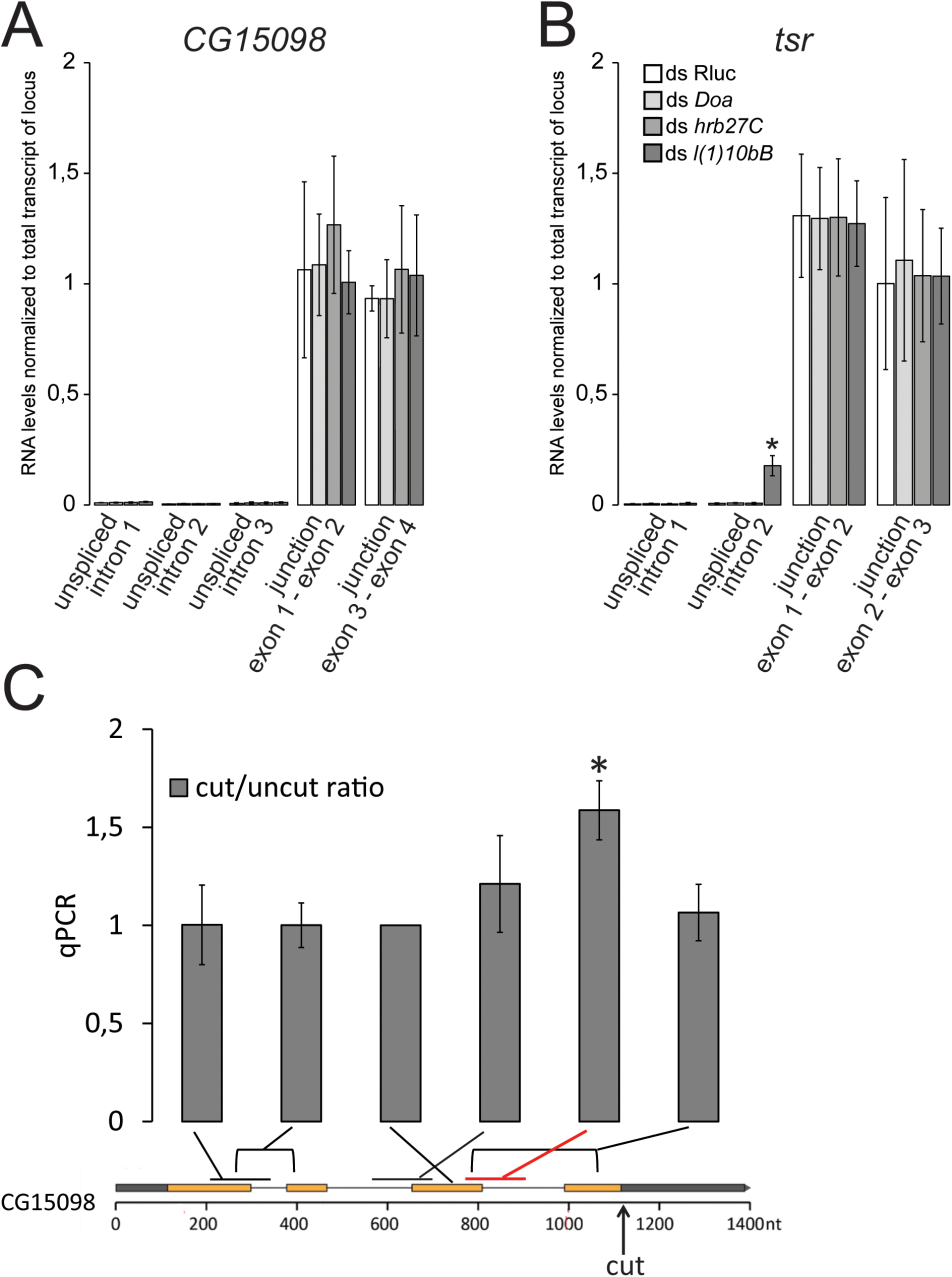
Knock-down of the screened splicing factors does not lead to a major decrease in overall splicing efficiency. (A) Following knock-down of a selected set of identified splicing factors, the levels of unspliced pre-mRNA were determined with amplicons that contained the intron-exon junction, the spliced messages were specifically amplified with amplicons where one primer spanned the exon-exon junction. An amplicon confined within an exon was used to normalize to the total amount of transcript produced at the locus. The values shown are the mean ± SD of three biological replicates.No increase of pre-mRNA levels was detected at any of the three introns in our CG15098 model locus and the levels of spliced transcript were comparable with the total transcript levels. (B) For the second intron of the *tsr* gene, we detected an increase of unspliced pre-mRNA after knock-down of *l(1)10Bb*. Nonetheless, the levels of spliced message were comparable to the total transcript levels, indicating that even in this case the majority of transcripts are still spliced. * *p* = 0.023, Student’s t-test (two-sided, n = 3 biological replicates) (C) Induction of a downstream DNA break slows CG15098 transcript maturation. We induced cleavage at the CG15098 lcous or in the TCTP gene as a control, isolated total RNA and performed qPCR analysis with random primed cDNA. The nascent RNA levels are much lower than those of mature CG15098 (see part A), but calculating the ratio of cut vs. uncut samples displays only the DNA-break induced changes. We detected a ~1.6-fold increase of nascent RNA directly upstream of the break (p<0.01, student’s t-test, 3 biological replicates) and there may be a trend towards slightly increased levels of nascent RNA further upstream at the second intron. We did not observe any changes in the level of mature CG15098 mRNA, which was to be expected given its far greater abundance and presumably slower turnover.

Since splicing is required to trigger the DNA-damage dependent siRNA response, we tested whether a knockdown of splicing factors identified in our screen simply reduces mRNA splicing and thereby diminishes the trigger for siRNA production. We thus determined splicing efficiencies at our CG15098 model locus and the *tsr* gene (both show strong expression in our S2 cells) following depletion of candidates recovered in our screen. After knockdown of the SR protein kinase *Doa*, the hnRNP protein *hrb27C* and the spliceosome component *l(1)10Bb* (the Bud31 homolog from the Prp19-related complex), we isolated total RNA and used qRT-PCR (random hexamer primed) to quantify the levels of unspliced pre-mRNA and spliced mRNA (Fig 4). The values were normalized to an amplicon that was internal to one of the exons and thus reported on the total amount of transcript from each locus (i.e. spliced and unspliced message). Overall, we found that the unspliced pre-mRNA did not increase relative to a control knockdown (*Renilla* luciferase). There was one exception, however: At the short intron in *tsr* the pre-mRNA became more abundant after RNAi against the spliceosome component *l(1)10Bb*. Even in this case, though, the amount of spliced message was comparable to the total amount of transcript produced, indicating that the majority of transcripts are correctly spliced. This was also seen for all other cases where we compared the level of spliced exon-exon junctions to the total amount of message, indicating that the canonically spliced mRNA accounts for essentially all of the transcripts detected at steady-state. In summary, we conclude that during the time of our knockdown-experiments there is no major change in general splicing efficiency. This argues that upon our experimental RNAi, the splicing factors and spliceosome components we identified did not yet induce major changes in mature mRNA levels. Rather than influencing the general cellular protein content, they may thus limit the signaling events that emanate from splicing reactions / spliceosomes perturbed by a nearby DNA break.

We used qRT-PCR to directly test whether a downstream DNA break perturbs progression of the splicing reaction. We induced cas9-mediated DNA cleavage downstream of the third intron of the CG15098 gene, then isolated total RNA and reverse transcribed both nascent and mature mRNA with random hexamer primers. We then interrogated nascent RNA with primers covering an exon-intron junction and mature mRNA with primers spanning an exon-exon junction. The samples were normalized to total CG15098 levels with an amplicon located inside of exon 3 as described above. Control samples were analyzed analogously and we calculated the cut/uncut ratio of each of the amplicons (Fig 4C). Indeed, we found significantly more unspliced RNA at the exon3-intron3 junction when the DNA was cut (student’s t-test p<0.01, 3 biological replicates). A downstream DNA break thus has the potential to stall progression of the splicing reaction at least transiently.

### At what stage is the splicing reaction stalled before double-stranded RNA is generated?

**Fig 5:**
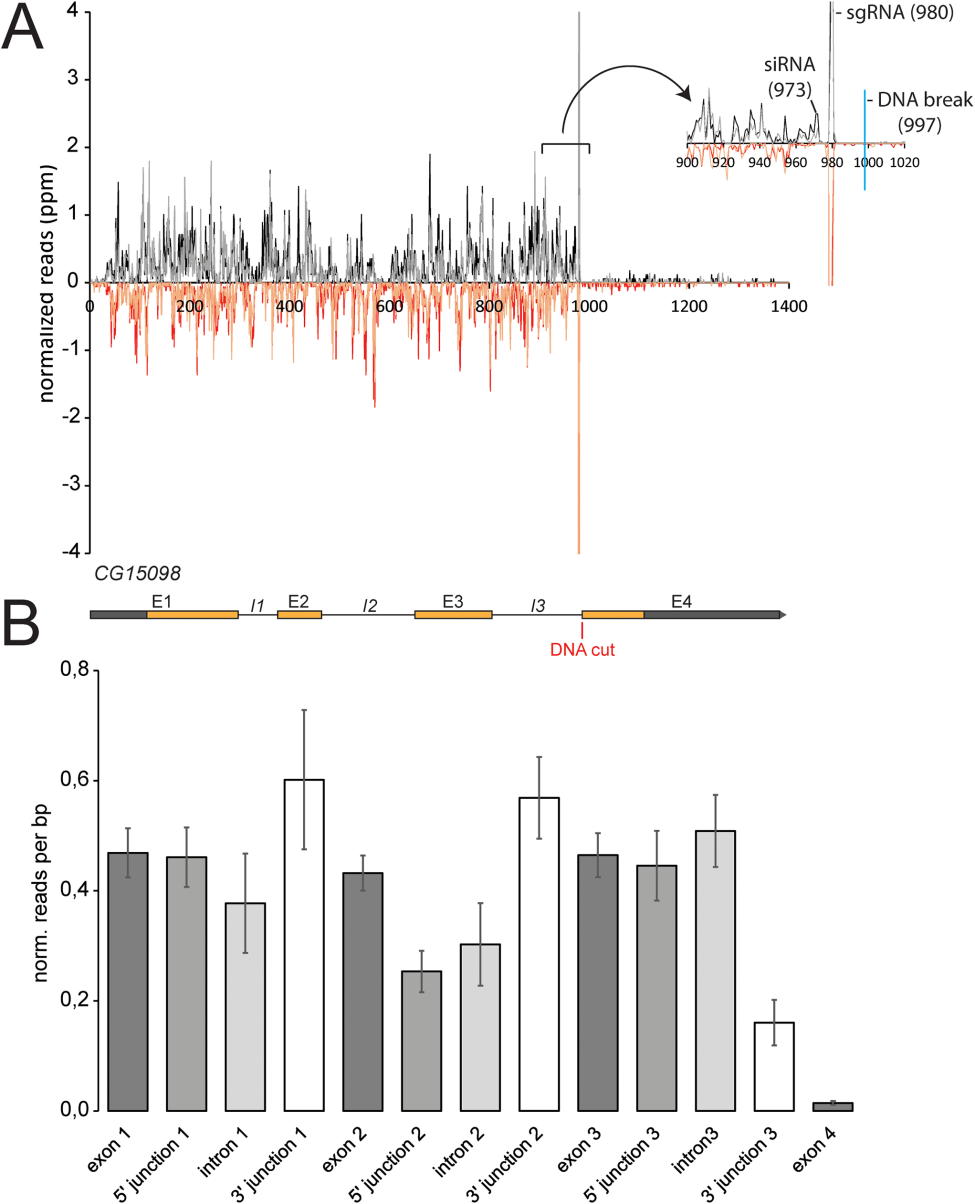
Detailed analysis of the siRNA coverage along the CG15098 gene. A set of four biological replicates was used to determine siRNA coverage along the gene. (A) Overall, the siRNA coverage was astonishingly reproducible. This diagram shows an overlay of two independent sequencing experiments (experiment 1 colored in red and black, experiment 2 colored in orange and gray), the traces indicate the position of the 5’-end for sense-oriented siRNAs and of the 3’-end for antisense-oriented siRNAs. The read coverage thus extends 21 nt downstream of the indicated position. A close-up view of the two traces is shown in the inset. (B) The reads covering individual exons, introns and the corresponding junctions were normalized to the size of the respective feature, then the average ± siRNA.

Since siRNA generation at DNA breaks depends on transcription levels in *Drosophila*, the sense strand of the siRNA precursor is most likely the normal transcript originating at the locus. Because upstream introns stimulate siRNA generation (Fig 3), we asked whether the splicing reaction takes place on the transcript molecule that contributes to the siRNA precursor. To this end, we analyzed the small RNA sequencing data from our cuts in the CG15098 gene in detail. The siRNA coverage started essentially adjacent to the induced breaks and continued relatively uniformly until the beginning of the CG15098 transcription start site (see e.g. S6 Fig). The pattern of siRNA coverage was astonishingly reproducible (Fig 5A), but there was no strong correlation between gene structure and local siRNA coverage (Fig 5B, calculated based on four biological replicates). Intron 2, but not intron 1 or 3, showed a somewhat lower coverage than the exons 1–3 (*p* = 0.016, two-sided Student’s t-test, n = 4 biological replicates). Exon-exon junction spanning siRNAs were essentially absent, consistent with the notion that the siRNAs are not generated by RdRP-activities acting on mature mRNA (*p*<10^−4^, S8 Fig). Furthermore, we found that the coverage of the 3’ intron-exon junctions–the site of the second transesterification reaction—was not diminished; rather, there was a trend towards slightly enhanced coverage relative to exonic reads (3’-junction 1: *p* = 0.11, 3’-junction 2: *p* = 0.06). Clearly, the sense transcript is not fully spliced prior to siRNA generation. If the splicing reaction is stalled after the first catalytic step, then siRNAs covering the 5’ exon-intron junction should be diminished. There was no change for intron 1 and 3 of CG15098. For intron 2 we observed a reduction of 5’ exon-intron spanning reads when compared with the exonic coverage (*p* = 2x10^-4^), but not when compared with the adjacent intron 2 coverage (*p =* 0,3). The reduced coverage for intron 2 as well as its 5’ splice junction remained when the DNA break was located at different positions relative to intron 2 (S9 Fig). We thus favor the interpretation that it is an inherent property of this intron (e.g. sequence-dependent). Because upstream splicing stimulates the siRNA response but the reaction is not completed, we conclude that the spliceosome is stalled most likely in a pre-catalytic state when dsRNA formation is triggered. This is also consistent with our qRT-PCR analysis (Fig 4C) where we saw an increase of nascent RNA as detected by an amplicon spanning the exon-intron junction, i.e. the site of the first catalytic step, after DNA cleavage.

## Discussion

The precursor of siRNAs in *Drosophila* is double-stranded RNA (dsRNA), which must be generated through convergent transcription since flies lack an RNA-dependent RNA polymerase. It was previously proposed that the DNA end serves as an initiation site for transcription that produces antisense RNA, followed by pairing with the normal transcript to generate dsRNA [16]. Our screening and validation experiments demonstrate that DNA end processing by the MRN-complex stimulates siRNA generation. Thus, recognition of the damage is independent of, and can precede, siRNA biogenesis. This is consistent with the observation that DNA damage signaling occurs normally in the absence of DNA damage induced siRNAs [14] and with the finding that DNA repair is unaffected when these siRNAs can no longer be made [22]. Our identification of the *Drosophila* CtIP homolog CG5872, a nuclease, in the screen further demonstrate that the generation of a 3’-single stranded DNA overhang facilitates the initiation of antisense transcription. Yet, siRNA coverage started adjacent to the break site, arguing that nucleolytic processing of the 5’-strand is not overly extensive. Based on our screening efforts we conclude that spliceosome components are required to trigger an siRNA response and cas9/CRISPR mediated cleavage in the genome revealed that an intron upstream of the break stimulates siRNA generation. The simplest interpretation is that spliceosomes participate in triggering this response independently of the splicing reaction. Consistently, we did not observe major splicing defects during the course of the knock-down experiments (Fig 4A and 4B), we could demonstrate that a downstream cut does slow down transcript maturation (Fig 4C) and the siRNA coverage analysis argued for a pre-catalytic stalling event (Fig 5).

**Fig 6:**
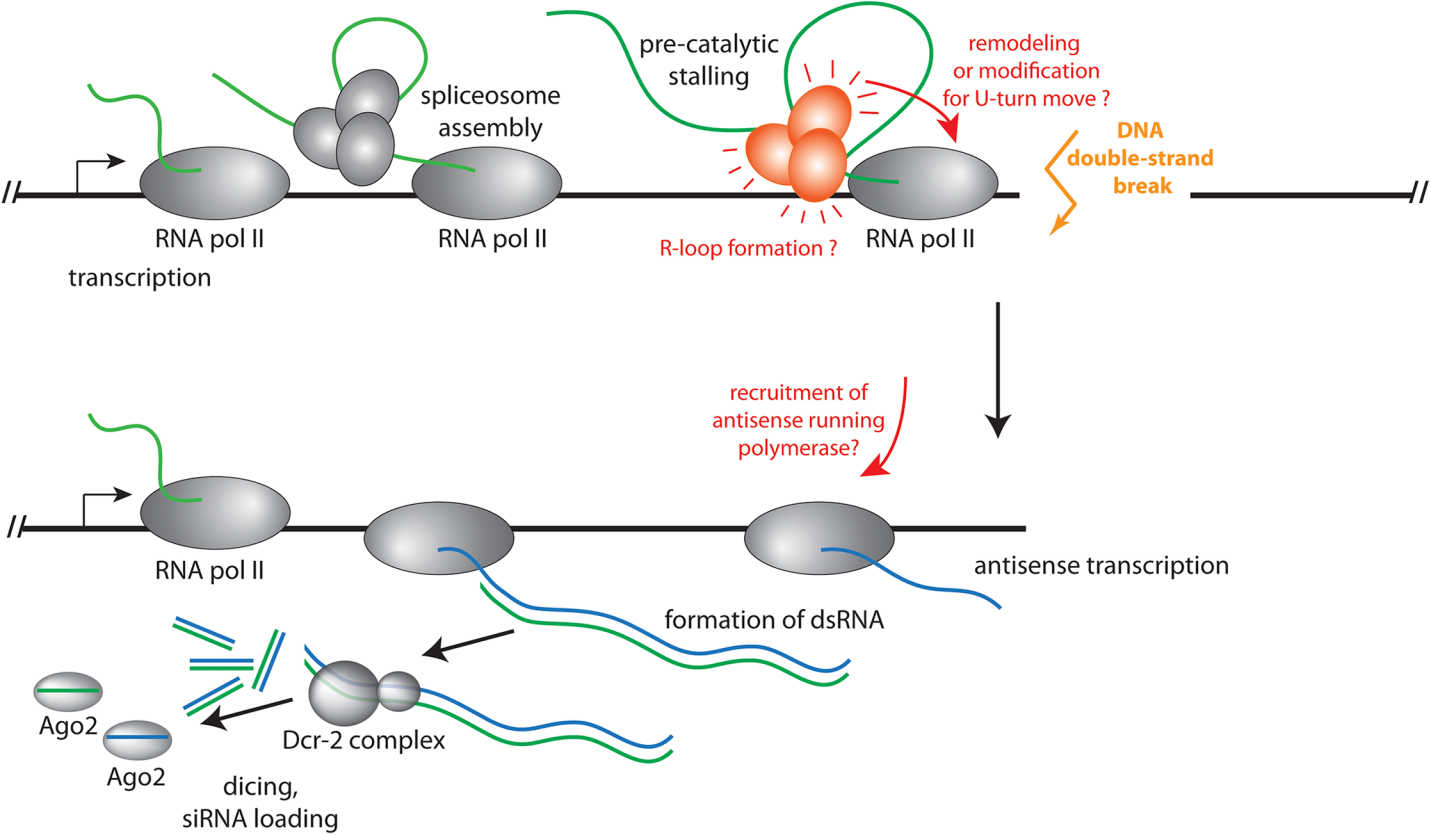
Model for the sequence of events leading to DNA DSB-induced siRNA formation in *Drosophila*. We propose that when the RNA polymerase reaches the DNA double-strand break, the co-transcriptional splicing process is adversely affected. This leads to a signaling event that emanates from the pre-catalytically stalled spliceosome, potentially augmented by formation of an R-loop, or a remodeling/modification of the RNA polymerase complex to enable a “U-turn” move and the synthesis of a long RNA hairpin. In both cases, double-stranded RNA is eventually produced when the normal mRNA transcript base-pairs with the break-induced antisense transcript. This double-stranded RNA is then converted into siRNAs by Dcr-2 and loaded into Ago2.

What is the mechanism of dsRNA generation at the break? A straightforward hypothesis is that upon reaching the broken DNA end, the transcribing RNA polymerase II simply turns around and continues transcription of the other DNA template strand, thus forming a hairpin transcript (“U-turn move”). This phenomenon is well known when DNA templates with protruding 3’-ends are transcribed by bacteriophage T7 RNA polymerase [29]. Similarly, free RNA polymerases could spontaneously initiate transcription at the newly formed DNA end; this is also readily observed *in vitro* with DNA templates that bear a 3’ single-stranded extension and was proposed to occur at DNA double-strand breaks in *S*. *pombe* [12]. In both cases, however, one would not predict that the presence of an intron should stimulate the generation of dsRNA; rather, DNA breaks in the intronless gene should have led to a comparable extent of siRNA generation as the ones in the intron-containing gene. Consistently, we had observed that transfected linear PCR products do not trigger an siRNA response [16]. Thus, while we cannot exclude that U-turn transcripts are formed, it is unlikely that they are the source of the majority of the siRNA precursors. It remains possible, however, that association of the spliceosome with the nascent mRNP leads to a remodeling or modification of the RNA polymerase complex, which favors the execution of a U-turn at the DNA end (see Fig 6). A formal possibility is that non-canonical enzymes are recruited to serve as RNA-dependent RNA polymerases (RdRP) acting on the transcript affected by the DNA break. For example, it has been demonstrated that RNA polymerase II can use an RNA template to create a corresponding RNA transcript in the case of human hepatitis delta virus or plant viroid replication [30–33] and that bacteriophage T7 RNA polymerase can replicate short RNA templates [34]. However, this has only been observed for RNAs with a particular secondary structure, while the DNA damage-induced siRNA response appears to be generic. Since we did not detect any exon-exon junction spanning reads in our siRNA coverage analysis, any RdRP-like activity would be limited to the unspliced, nascent transcript. Taken together, we do not consider this a likely scenario. The most parismonious hypothesis is that RNA polymerase stalls at the break, co-transcriptional mRNA maturation is concomitantly delayed and that this induces a signaling event with participation of the spliceosome (Fig 6). Such a stalled transcript probably leads to a persistent R-loop with a corresponding displaced, single-stranded DNA region. This single-stranded DNA, together with a signal from the spliceosome, could serve as an initiation site for antisense transcription. It is also conceivable that such R-loops may be larger or more persistent if the stalling occurs in the vicinity of an engaged spliceosome without the need for a specific signaling step. This mechanism may also act when R-loops occur independently of a DNA break, consistent with the observation that the splicing factors we identified were also required for the small RNA response triggered by high-copy transgenes.

Several previous publications have reported a requirement of splicing factors, but not splicing in general, for small RNA-mediated transcriptional silencing in fission yeast [35, 36]. Consistent with this, centromeric non-coding transcripts are indeed spliced in *S*. *pombe* [36]. However, messages coding for essential fission yeast silencing factors appear to be particularly sensitive to diminished splicing activity, thus leading to reduced silencing efficiency; a similar phenomenon can affect the expression of the *Drosophila melanogaster* piRNA factor *piwi* [37, 38]. It remains a matter of debate whether intron-less, cDNA-based rescue constructs can bypass the silencing defect induced by perturbed splicing in *S*. *pombe* [36, 37]. Other splicing factors, such as *smD1*, appear to function independently of their splicing activity during miRNA and siRNA RISC formation [39, 40]. We now demonstrate that for DNA double-strand break triggered siRNAs, an intron is required upstream (with respect to transcription) of the lesion to trigger siRNA formation (Fig 3). This strongly supports the notion that the splicing process acts in *cis* during siRNA generation rather than *in trans via* perturbed splicing of silencing factor mRNAs.

Based on our siRNA coverage and qRT-PCR analysis, we propose that the spliceosome is stalled at a pre-catalytic stage (see the model in Fig 6). The Prp19 complex promotes the transition into the catalytic splicing phases and members of this complex appeared enriched among the spliceosome components we identified (Fig 2D and S3 Fig). Prp19 is of central importance for the splicing-mediated identification of transposon transcripts in *C*. *neoformans* [27], but in this case the reaction was stalled after the first catalytic step. The *Drosophila* response we describe is conceptually similar to the recently discovered spliceosome-mediated decay in the budding yeast *Saccharomyces cerevisiae*. There, nucleases are recruited to intron-less genes as a consequence of non-productive association with the spliceosome [41]. Splicing controls piRNA biogenesis in fruit flies; this is a class of small RNAs that represses–together with endo-siRNAs–transposable elements in the germline. Here, spliced transcripts from the so-called master control loci are prevented from entering the piRNA biogenesis pathway [42]. On the other hand, the Tho/TREX complex, normally deposited on RNA as a consequence of splicing, is essential for piRNA biogenesis and must associate through an alternative route with unspliced piRNA precursors [43]. Interestingly, we identified the Tho complex component *tho2* as a potential inhibitor of both, DNA damage-induced siRNAs as well as high-copy transgene induced siRNAs (S1 Table).

Induction of DNA damage by UV-light revealed that in human cells, splicing is both a sensor for as well as a target of the DNA damage response. This depends on the formation of R-loops due to stalled polymerases and results in pleiotropic splicing changes. The phenomenon bears many parallels to our analysis, but it was limited to transcription-blocking lesions and the authors specifically excluded DNA double-strand breaks as triggers [44]. A consequence of R-loop formation is the generation of a corresponding stretch of displaced, single-stranded DNA. Potentially, this DNA is covered by replication protein A (RPA), [45], a situation that can trigger DNA damage signaling via direct interaction with Prp19 and accumulation of ATR-interacting protein (ATRIP) in mammalian cells [13]. This role of Prp19 appears to be independent of its function during the splicing reaction. Future experiments should therefore address the question whether multiple recruitment platforms rely on the conversion of the Prp19 complex from a regulator of the splicing reaction into a trigger for DNA damage associated signaling events.

Due to the focus on siRNA biogenesis in our screen, rather than their downstream function, we cannot directly conclude on the benefits of the break-derived siRNAs for the organism. We previously demonstrated that *dcr-2* and *ago2* play–at best–an accessory role during DNA repair [22], but this only addresses the importance of the siRNAs and not their precursor molecules. It is possible that conversion of the stalled transcript into double-stranded RNA or the process of antisense transcription *per se* are important for DNA repair. For example, this could limit the extent of R-loop formation behind a stalled RNA polymerase (both temporally and spatially) or set the optimal length of RPA-covered single-stranded DNA, as was recently proposed for *Schizosaccharomyces pombe* [12]. Control of R-Loop size during transcription has also been described: In the centromeric regions of fission yeast, a specific arrangement of replication origins and repetitive elements leads to frequent collisions of an RNA polymerase and the replication machinery, followed by the generation of siRNAs in an RdRP-dependent process. In this case, RNA interference prevents excessive R-loop formation by releasing RNA polymerase II and thus fosters genome integrity [46]. Other publications have described a role for RNAi during DNA repair in other organisms [14, 15, 18, 19, 47]. In the case of the damage-induced *Neurospora crassa* qiRNAs, DNA repair was even proposed to be the trigger for small RNA production [19]. We note that in the case of our model Gene CG15098, no repetitive and thus recombination-favoring sequence arrangement was required. Clearly, more results are needed to delineate common and divergent features between these experimental systems. Since the splicing-dependent surveillance mechanism is also important for siRNA generation from high-copy transgenes (Fig 2B), it is possible that the DNA double-strand breaks fortuitously trigger the splicing-dependent mRNA maturation check-point in *Drosophila melanogaster*, while its primary importance might be to act as a surveillance mechanism against foreign DNA sequences. The list of candidates we identified in our screen provides a valuable resource to further our understanding of the molecular mechanisms and the biological significance of this surveillance mechanism.

## Materials and methods

### Cell culture, transfections and cas9-CRISPR mediated induction of DNA double-strand breaks

Cells were cultured and transfected as described [48]. DNA double-strand breaks were induced in an S2-cell clone that stably expresses the cas9 nuclease [49] with linear U6-sgRNA fusion fragments generated by overlap extension PCR as described [50]. The primer sequences for sgRNA expression are provided in S2 Table. The cell line with stable high-copy integration of lucifcerase reproters was generated by transfecting S2-cells with a mix containing pRB1 (Renilla luc.) in a 10-fold excess over pRB2 (firefly luc.) and phsHygro. After selection of stable resistance the cells were diluted, clones were picked and clone 4 was used for the experiments.

### Genome-wide RNAi screen

A suspension of DMel cells (an S2-cell isolate) in Express5 SFM was prepared (final concentration of 25.000 cells/well) and 30 μL cell suspension were added to each well of a Greiner 384-well plate containing 5 μL of dsRNA (conc. 50ng/μL). The plates were sealed and incubated at 25 C for 48 h. On each plate of the screen, we included a set of positive controls (knock-down of Ago2, Dcr-2 and Ago1) as well as a set of negative controls (knock-down of GFP and DsRed). Furthermore, we included a knock-down of *Renilla* luciferase as a positive control for potential repressors of DSB-induced siRNA generation. Finally, we knocked down the apoptosis inhibitor *thread* to set thresholds for exclusion of data from predominantly dead cells.

For transfection, 0.15 μl/well Fugene (Promega) and 6 ng/well of pRB1, 20 ng/well of pRB2 and 30 ng/well of linearized pRB3 were pre-diluted in 14.85 μl/well Express5 SFM and incubated at room temperature for 30 min. 15 μL of the thus prepared transfection solution were then dispensed into each well of the assay 384-well plates containing 35 μL of cell suspension with dsRNA. The plates were again sealed and incubated at 25°C.

96 h after knockdown, cells were lysed and luminescence was measured. Fluc and Rluc buffers were added subsequently to the cell lysate and the signals were measured 5 min after the addition of the according substrate with no filter (Fluc) and a F485 Coelenterazin filter (Rluc).

The primary analysis of the FLuc and RLuc data that was obtained from luminescence measurements was done in R using the cellHTS2 package [51]. The raw data was log-transformed and normalized on the plate median, each channel was analyzed separately and no variance adjustment was applied. The mean RLuc values were plotted against the mean FLuc values and a smoothened curve was fitted to the data using locally weighted polynomial regression (LOESS) with a smoothing parameter of 0.9. The thus predicted LOESS values were subtracted from the corresponding mean RLuc values to obtain the LOESS-residuals (resi). The residuals were then used to calculate a z-score. A comprehensive overview of the screening data and controls is given in S2 Fig, the original screening data has been deposited at http://www.genomernai.org.

### Validation experiments

For validation, S2 cells (lab stock) were grown in FBS-supplemented Schneider’s medium and the validation screens were conducted in 96-well plates with linearized pRB4 as siRNA inducer. The knockdown constructs used for validation are provided in S1 Table.

The oligonucleotide sequences for the generation of the independent dsRNA constructs for validation are provided in S1 Table. *In vitro* transcription and treatment of the cells was as previously described [16].

### Generation and analysis of next generation sequencing data for the characterization of DSB-induced siRNAs

A total volume of 3 ml of stable cas9-expressing cell culture was transfected with 1500 ng of U6-sgRNA PCR product as described [50]. Five days after transfection, total RNA was isolated using Trizol and deep sequencing libraries were generated [52]. In certain cases, two sgRNAs were introduced, leading to cas9-mediated cleavage in distinct genes. Up to four libraries were barcoded, combined and analyzed on one lane of an Illumina HiSeq instrument. Subsequently, the obtained reads were sorted, trimmed and mapped to the regions of interest using bowtie [53]. Further analysis was performed with in-house Perl scripts (available on request).

### Quantitative RT-PCR

Cells were treated with dsRNA to induce knock-down of the corresponding genes under identical conditions as for screening and validation. Five days after induction of knock-down, total RNA was isolated using Trizol and the RNA was reverse transcribed with random hexamer primers and the Superscript-III enzyme. For the analysis of splicing upstream of a DNA break, we transfected the corresponding sgRNA expression PCR products into our stable cas9-expressing cell line, then isolated total RNA 3 days after transfection. Quantitative PCR was carried out on a Biometra TOptical real-time PCR cycler with the Dynamo Flash SybrGreen PCR kit (Finnzymes). Data analysis was done according to the 2^-ΔΔCt^ method [54], primer sequences for qPCR are given in S2 Table.

## Supporting information

S1 Fig The figure depicts the workflow of our genome-wide screen for DNA double-strand break induced siRNA biogenesis. Note that the promoters driving expression of firefly (tubulin) and *Renilla* (ubiquitin) luciferase both contain an intron in the 5’-UTR. Following the screen, stringent validations were performed with two independent sets of dsRNA triggers and only those candidates were retained that scored positive in two out of three dsRNA designs (bottom diagram).
(JPG)

S2 Fig This diagram gives a comprehensive overview of all the screening data (including the positive and negative controls). (JPG)

S3 Fig This figure presents a comprehensive map of the spliceosome components and complexes that assemble along the splicing reaction. The assignment is based on the information contained in the spliceosome database (http://spliceosomedb.ucsc.edu/proteins) as of June 2016. Please note that assignment of individual genes to a specific complex may not always be unambiguous but had to be simplified for this diagram. Furthermore, this map is not absolutely comprehensive as some simplification and consolidation was necessary to preserve clarity. The color code depicts the effect strength in the original screening data (Z-score, corresponding numerical values can be found in S1 Table). The effect strength for a knock-down of Dcr-2 is shown as a reference below the color key.
(JPG)

S4 Fig Assessment of cleavage and mutagenesis at the cas9-CRISPR target sites. We performed a T7 endonuclease assay on PCR products obtained from DNA isolated alongside with the RNA for our deep sequencing experiments. Processing by T7 endonuclease at the intended position is indicated with an asterisk and demonstrates that the corresponding site had been cleaved and subject to mutagenic repair *in vivo*. The results are grouped according to cleavage within CG15098 (A), TCTP (B) or CG18273 (C).
(JPG)

S5 Fig Detailed traces of siRNA reads mapping to the intronless *tctp* gene. Examples for the two positions of cas9-CRISPR mediated cuts in the intronless *tctp* gene are depicted; the position of the cut site can be deduced from the large peak that derives from the targeting region of the sgRNA itself. Please refer to Fig 3 of the manuscript for the correlation of cleavage site with gene structure.
(JPG)

S6 Fig Detailed traces of siRNA reads mapping to the intron-containing *CG15098* gene. A selection of cas9-CRISPR mediated cuts are depicted; The position of the cut site can be deduced from the large peak that derives from the targeting region of the sgRNA itself. Please refer to Fig 3 of the manuscript for the correlation of cleavage site with gene structure.
(JPG)

S7 Fig Detailed traces of siRNA reads mapping to the intron-containing *CG18273* gene. The blue line indicates the position of the cas9-mediated cut. Note the scale change relative to the previous figure.
(JPG)

S8 Fig Analysis of exon-intron and exon-exon spanning siRNA coverage. We used a dataset with four replicate experiments containing a cut at the end of the third intron in the CG15098 gene (CRISPR 207). All exons, introns and junctions that lie upstream of the cut were combined and an average ± SD for the four replicates was calculated. In addition, we mapped the sequencing data to a CG15098 cDNA sequence, and then calculated the abundance of exon-exon junction spanning reads in our data.
(JPG)

S9 Fig Analysis of read coverage according to gene structure. (A) For data derived from a cut within the 3’-UTR of CG15098 (CRISPR 750); (B) For data derived from a cut within the third exon (CRISPR 749).
(JPG)

S1 Table Detailed information on the candidate validation experiments. (XLSX)

S2 Table Primer sequences for sgRNA+T7 endonuclease assays and primer sequences for qPCR. (XLSX)
