# Supplementary figures and images for: Splicing stimulates siRNA formation at Drosophila DNA double-strand breaks
Source: PLoS Genet. 2017 Jun 19;13(6):e1006861. doi: 10.1371/journal.pgen.1006861 (PMC5495518; doi:10.1371/journal.pgen.1006861)

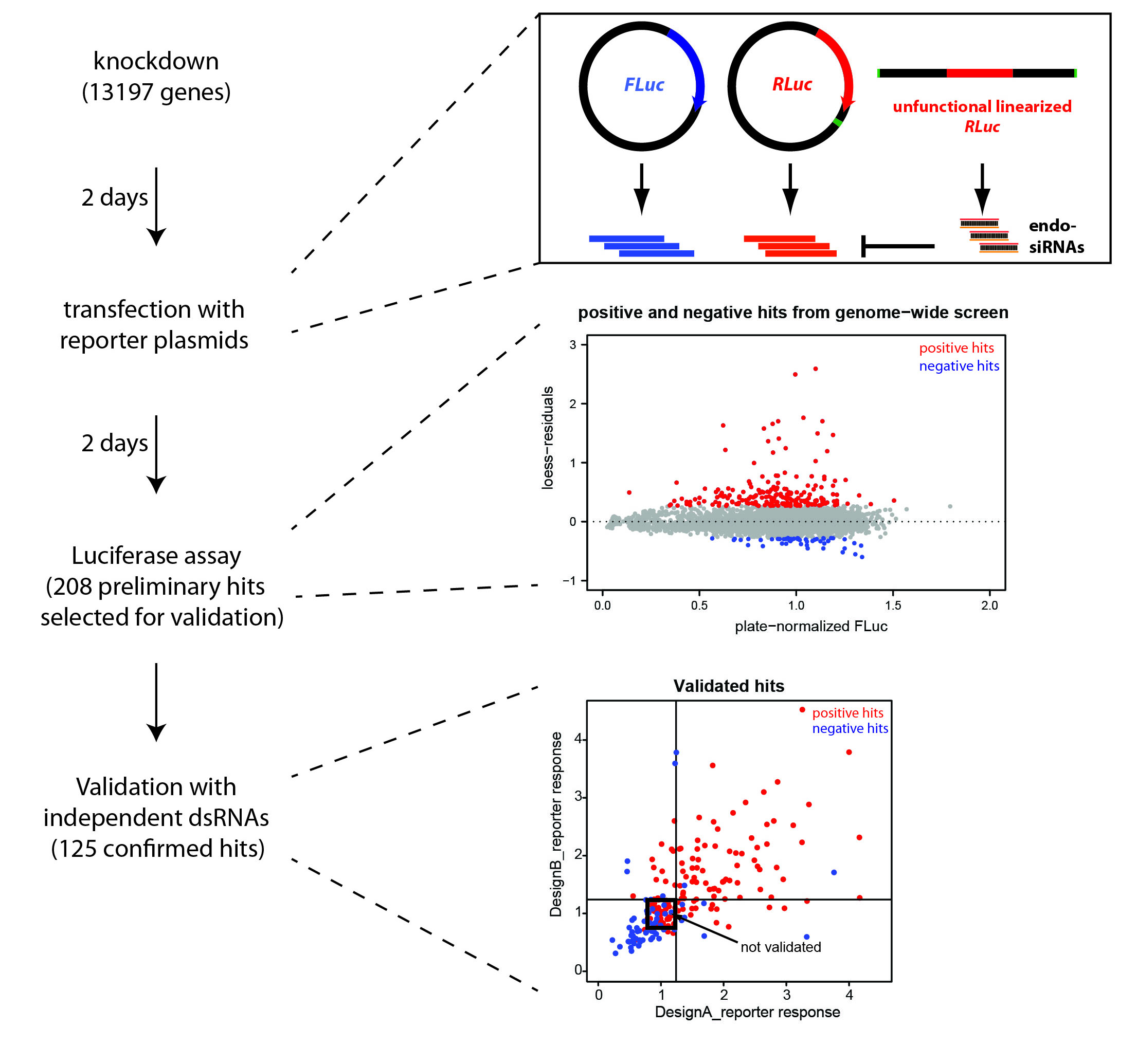

Supplement: S1 Fig — Note that the promoters driving expression of firefly (tubulin) and Renilla (ubiquitin) luciferase both contain an intron in the 5’-UTR. Following the screen, stringent validations were performed with two independent sets of dsRNA triggers and only those candidates were retained that scored positive in two out of three dsRNA designs (bottom diagram). (JPG) [file pgen.1006861.s001.jpg]

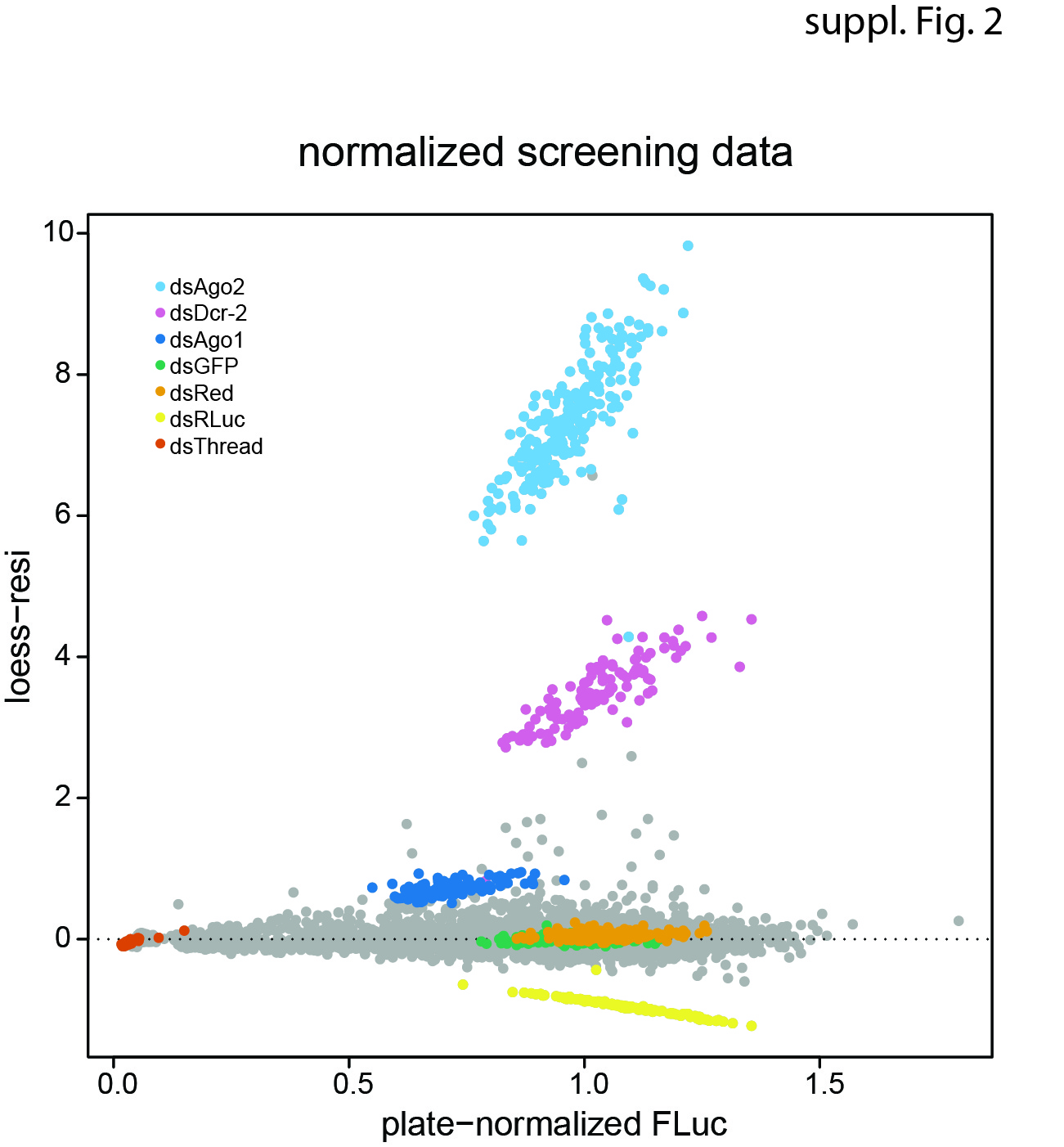

Supplement: S2 Fig — (JPG) [file pgen.1006861.s002.jpg]

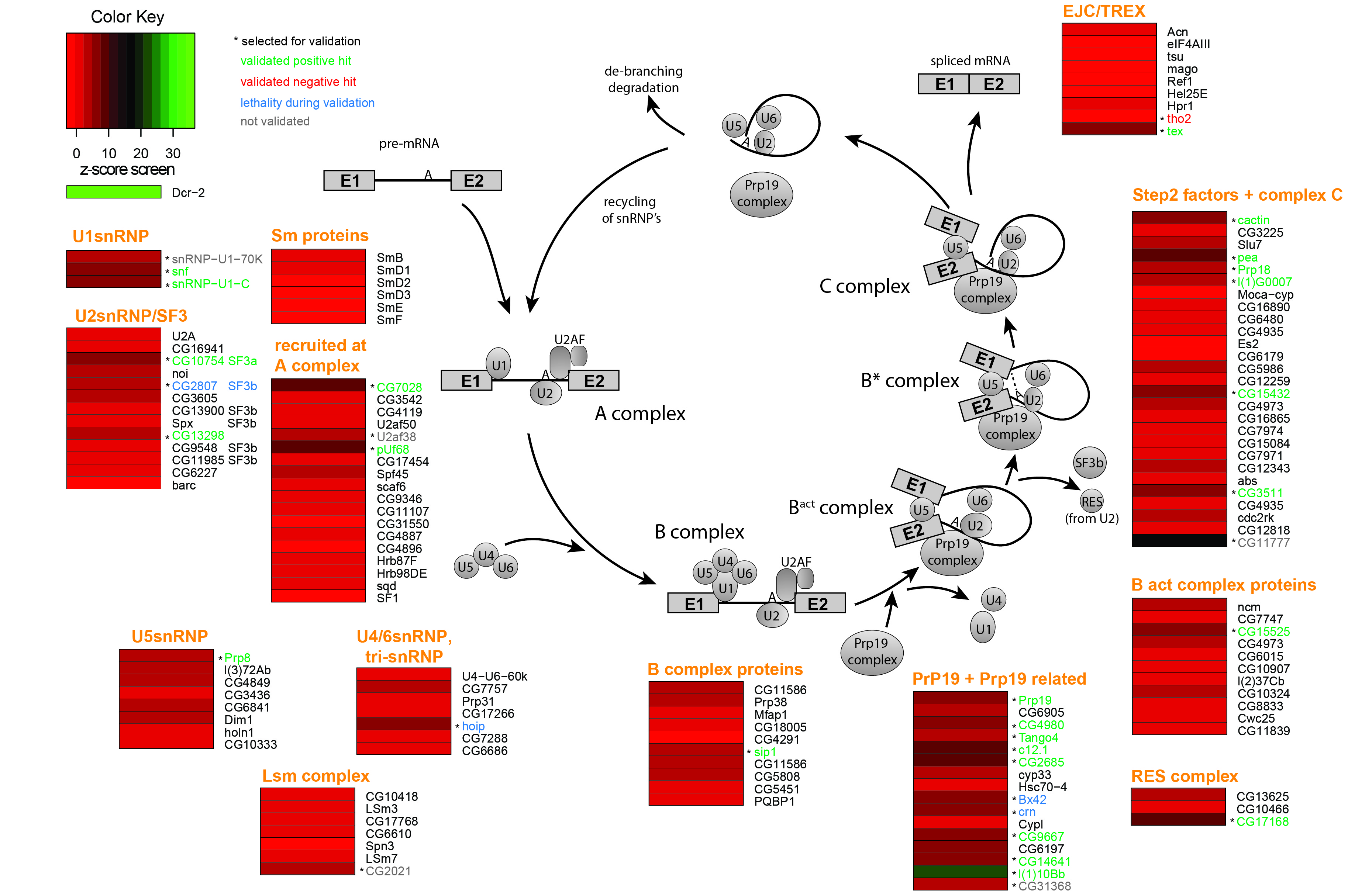

Supplement: S3 Fig — The assignment is based on the information contained in the spliceosome database (http://spliceosomedb.ucsc.edu/proteins) as of June 2016. Please note that assignment of individual genes to a specific complex may not always be unambiguous but had to be simplified for this diagram. Furthermore, this map is not absolutely comprehensive as some simplification and consolidation was necessary to preserve clarity. The color code depicts the effect strength in the original screening data (Z-score, corresponding numerical values can be found in S1 Table). The effect strength for a knock-down of Dcr-2 is shown as a reference below the color key. (JPG) [file pgen.1006861.s003.jpg]

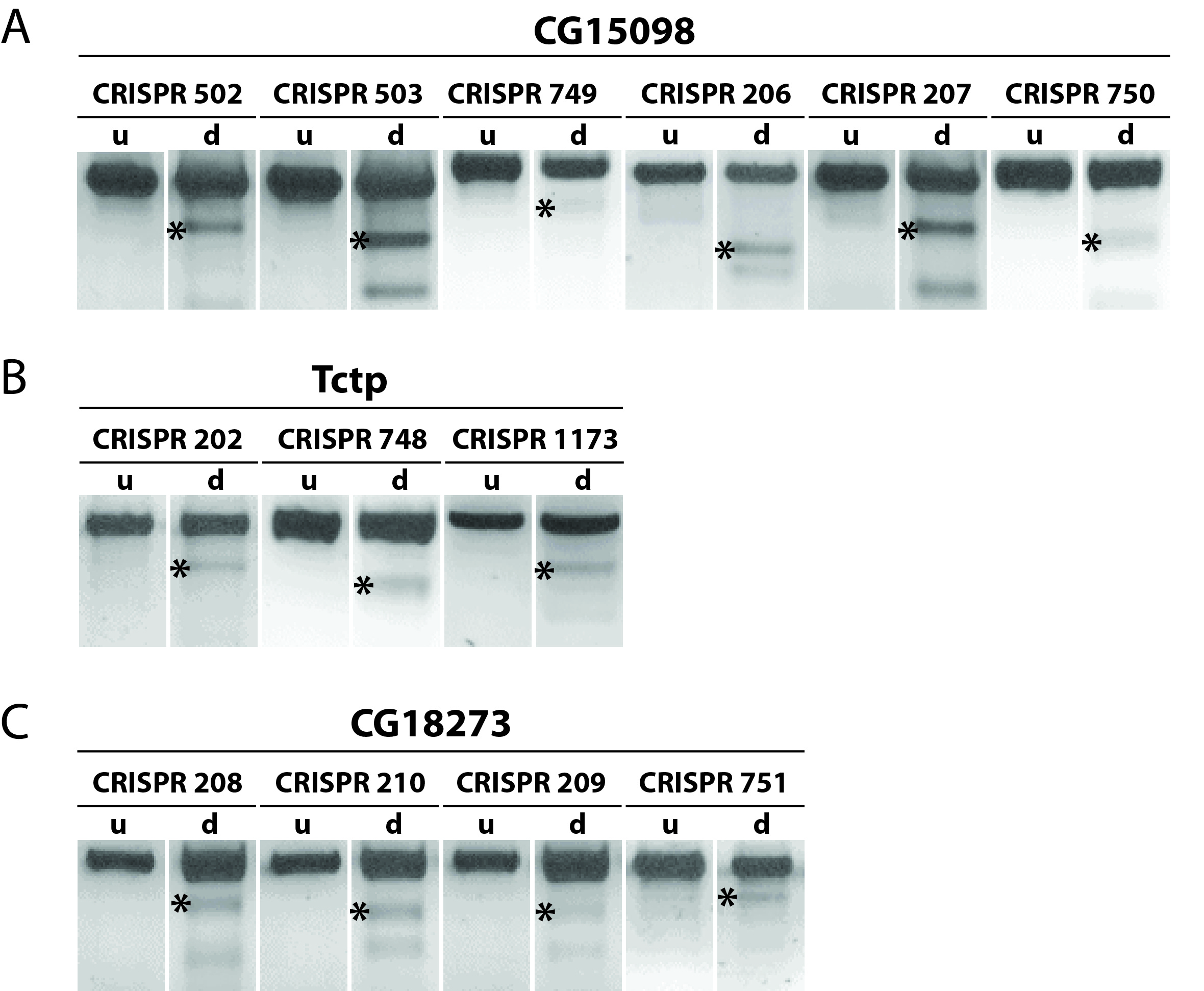

Supplement: S4 Fig — We performed a T7 endonuclease assay on PCR products obtained from DNA isolated alongside with the RNA for our deep sequencing experiments. Processing by T7 endonuclease at the intended position is indicated with an asterisk and demonstrates that the corresponding site had been cleaved and subject to mutagenic repair in vivo. The results are grouped according to cleavage within CG15098 (A), TCTP (B) or CG18273 (C). (JPG) [file pgen.1006861.s004.jpg]

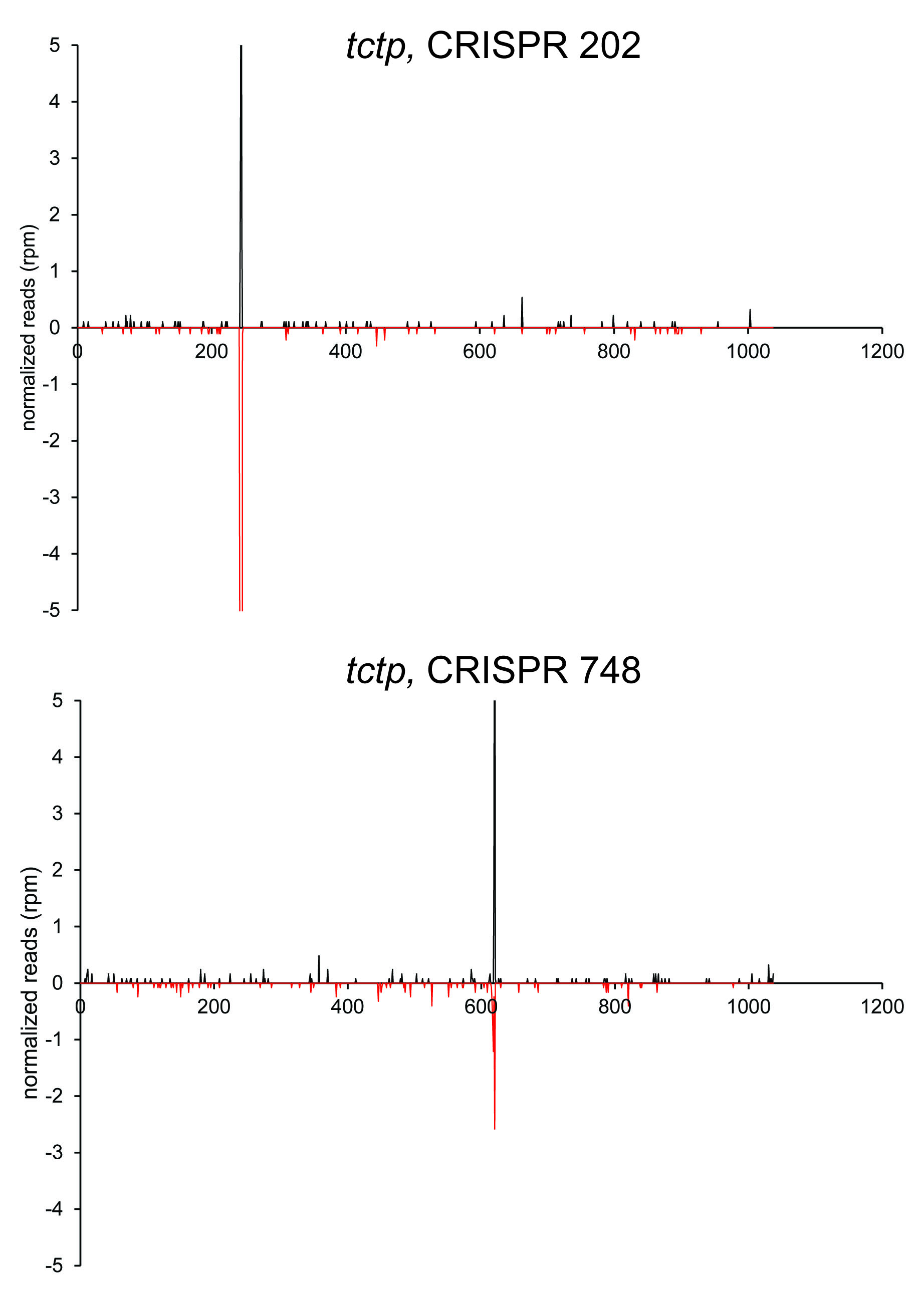

Supplement: S5 Fig — Examples for the two positions of cas9-CRISPR mediated cuts in the intronless tctp gene are depicted; the position of the cut site can be deduced from the large peak that derives from the targeting region of the sgRNA itself. Please refer to Fig 3 of the manuscript for the correlation of cleavage site with gene structure. (JPG) [file pgen.1006861.s005.jpg]

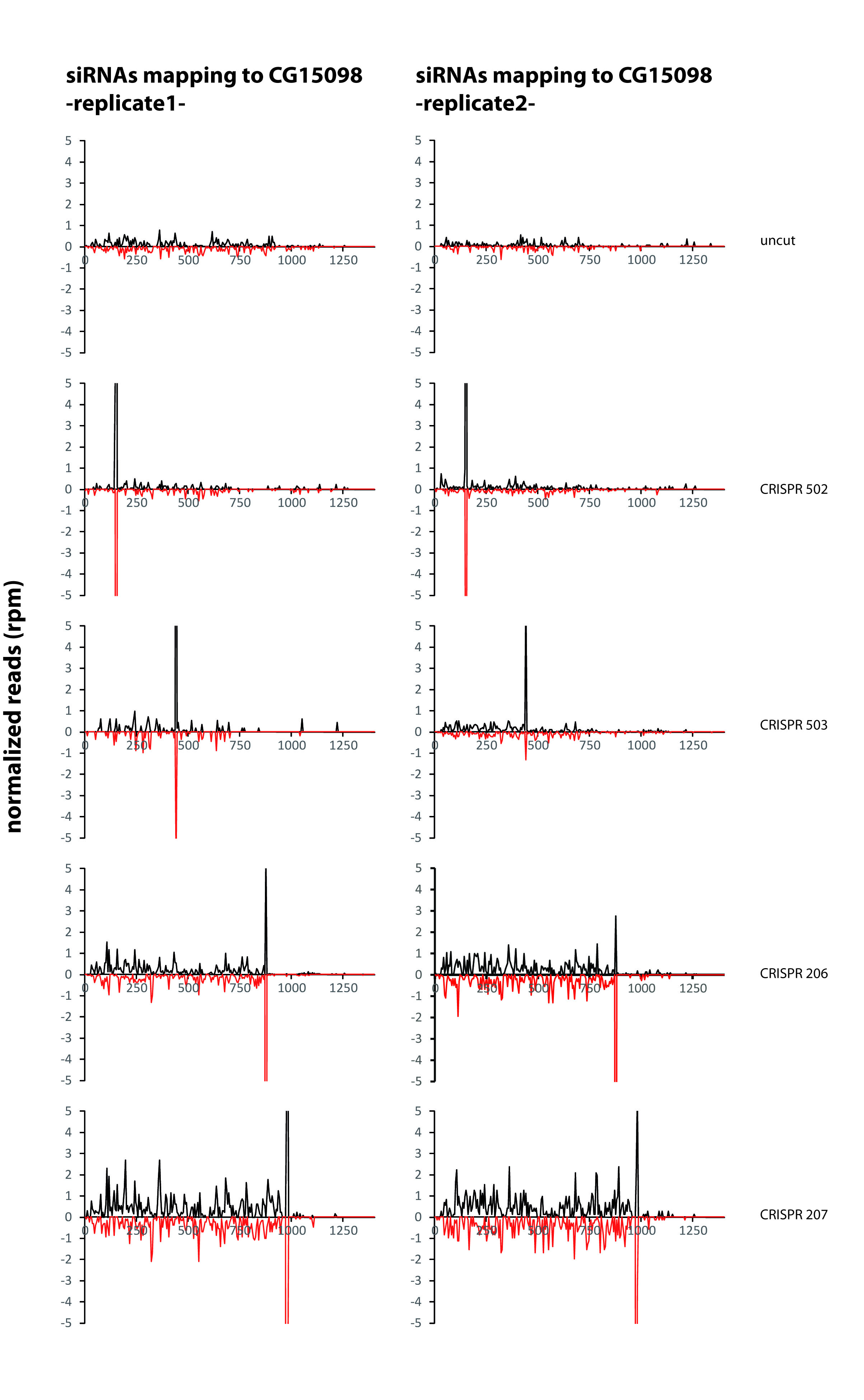

Supplement: S6 Fig — A selection of cas9-CRISPR mediated cuts are depicted; The position of the cut site can be deduced from the large peak that derives from the targeting region of the sgRNA itself. Please refer to Fig 3 of the manuscript for the correlation of cleavage site with gene structure. (JPG) [file pgen.1006861.s006.jpg]

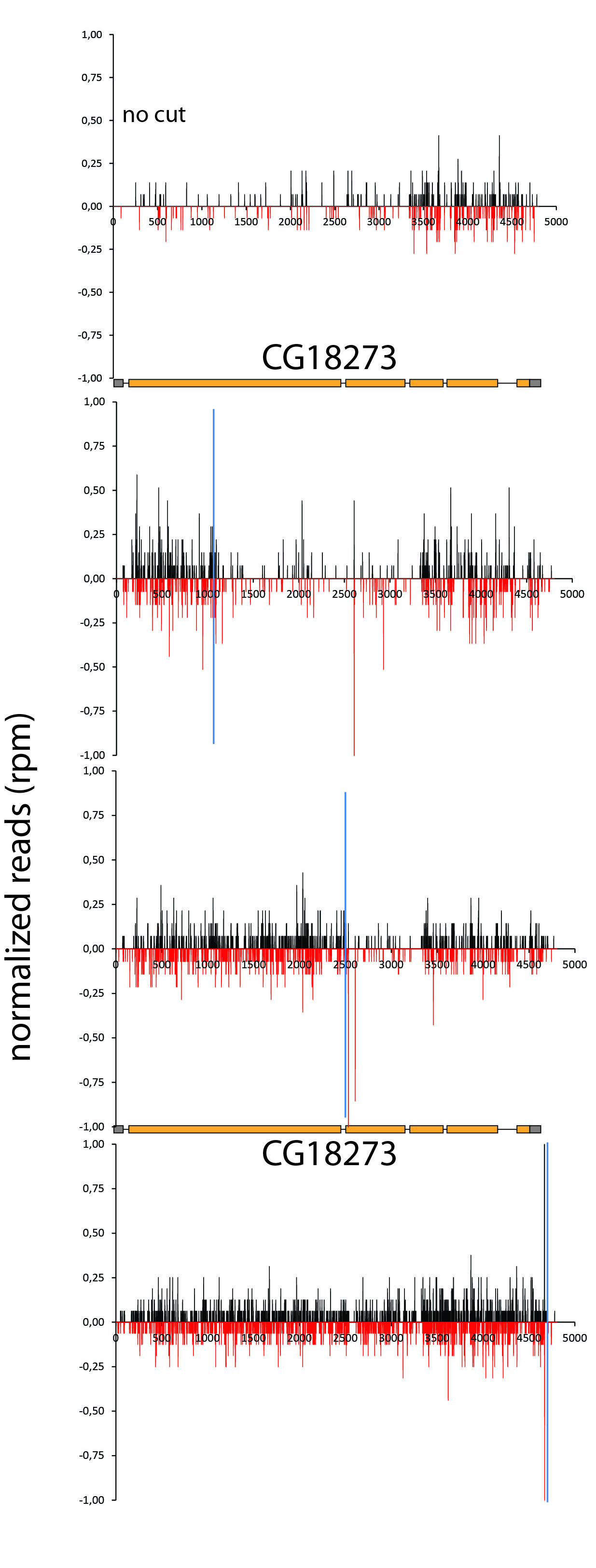

Supplement: S7 Fig — The blue line indicates the position of the cas9-mediated cut. Note the scale change relative to the previous figure. (JPG) [file pgen.1006861.s007.jpg]

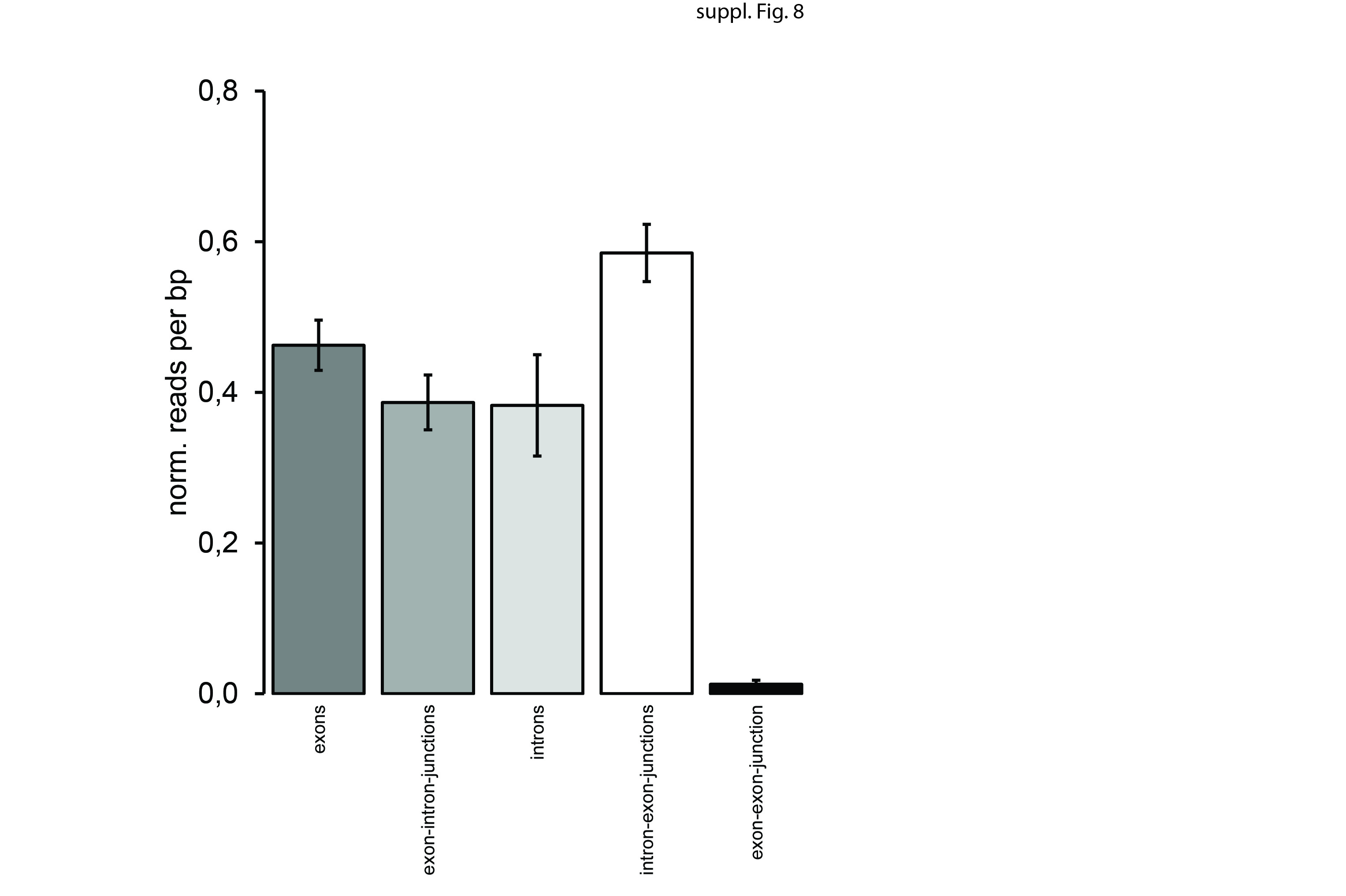

Supplement: S8 Fig — We used a dataset with four replicate experiments containing a cut at the end of the third intron in the CG15098 gene (CRISPR 207). All exons, introns and junctions that lie upstream of the cut were combined and an average ± SD for the four replicates was calculated. In addition, we mapped the sequencing data to a CG15098 cDNA sequence, and then calculated the abundance of exon-exon junction spanning reads in our data. (JPG) [file pgen.1006861.s008.jpg]

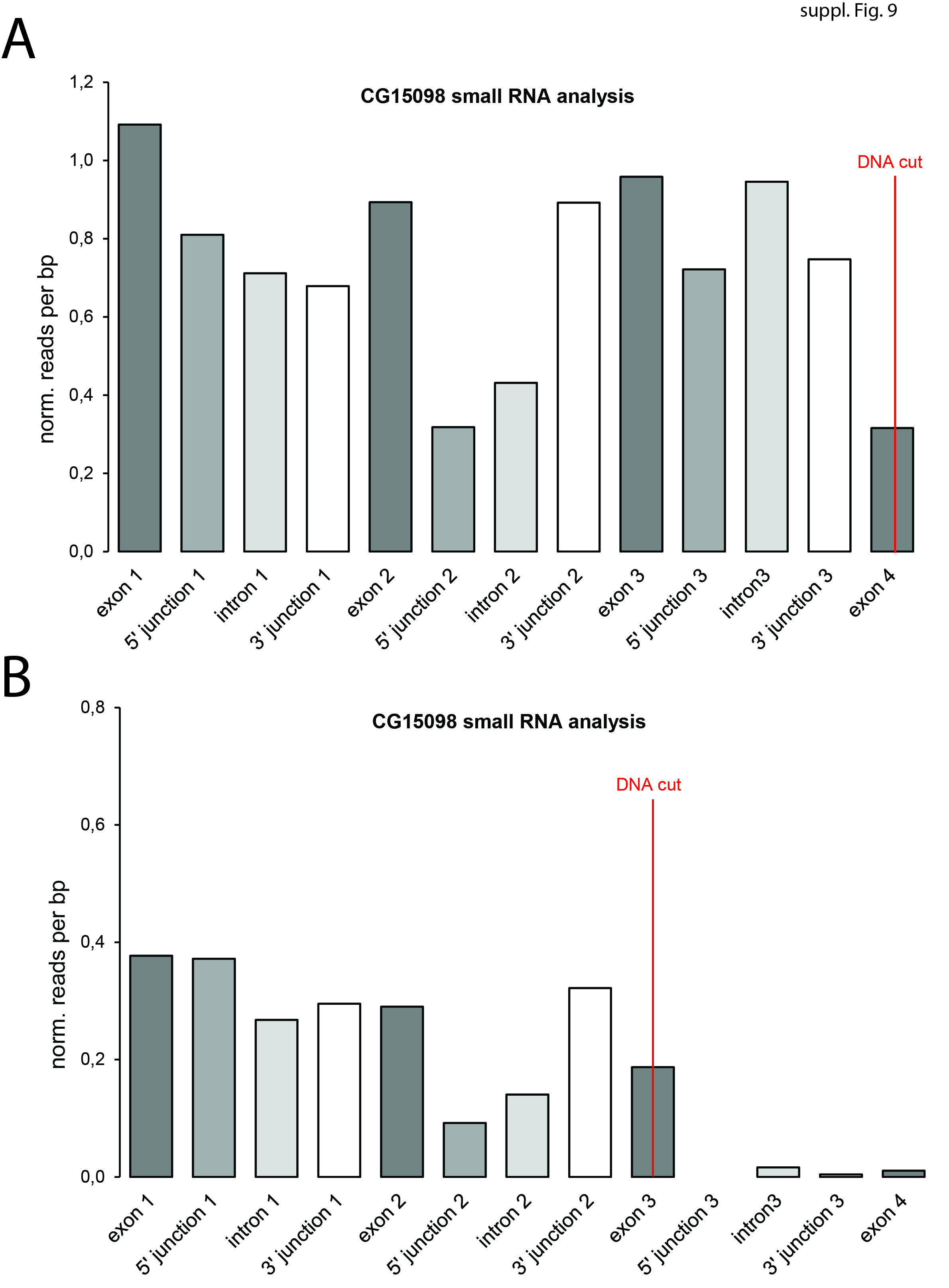

Supplement: S9 Fig — (A) For data derived from a cut within the 3’-UTR of CG15098 (CRISPR 750); (B) For data derived from a cut within the third exon (CRISPR 749). (JPG) [file pgen.1006861.s009.jpg]
